# Examining the nature of feedback within the Mini Clinical Evaluation Exercise (Mini-CEX): an analysis of 1427 Mini-CEX assessment forms

**DOI:** 10.3205/zma001193

**Published:** 2018-11-15

**Authors:** Diantha Soemantri, Agnes Dodds, Geoff Mccoll

**Affiliations:** 1 Faculty of Medicine Universitas Indonesia, Department of Medical Education, Jakarta, Indonesia; 2 University of Melbourne, Melbourne Medical School, Department of Medical Education, Melbourne, Australia; 3 University of Queensland, Faculty of Medicine, Executive Dean, St. Lucia Queensland, Australia

**Keywords:** feedback, clinical, assessment

## Abstract

**Background: **Despite the growing use, studies have demonstrated some limitations related to the feedback provided in the context of the increasing use of the Mini Clinical Evaluation Exercise (Mini-CEX) in undergraduate medical education. This study examined the written feedback provided on the Mini-CEX form to determine its usefulness as a learning tool for students.

**Methods: **1427 Mini-CEX assessment forms of final year medical students were collected. Written feedback, both on students’ strength and weakness, was categorized and correlated with the variables of clinical case complexity, assessors’ clinical position and students’ clinical performance rating.

**Results:** The number of general feedback comments for students’ strengths and development were 953 (65.3%) and 604 (38.64%) respectively. Less than 30% of the feedback for each domain was categorized as specific feedback. Significant associations were found between feedback on strength and clinical case complexity (Χ^2^=17.48, *p*<.01); and also with assessor clinical position (Χ^2^=37.10, *p*<.01). There was also an association between feedback for students’ development and assessor clinical position (Χ^2^=27.22, *p*<.01).

**Conclusion:** Based on the Mini-CEX forms of student cohort this study examined, it can be concluded that the written feedback provided in the Mini-CEX assessment form was general and lacked specificity. This finding leads to the need to train clinical teachers in the provision of feedback.

## Zusammenfassung

**Hintergrund:** Trotz steigender Anwendungszahlen der Mini Clinical Evaluation Exercise (Mini-CEX) Prüfung, die in der medizinischen Grundausbildung zunehmend genutzt wird, haben Studien einige Einschränkungen im Zusammenhang mit dem abgegebenen Feedback (Rückmeldung) gezeigt.

Diese Studie überprüft das schriftlich abgegebene Feedback auf Mini-CEX-Formularen, um die Nutzbarkeit dieser als Lernhilfsmittel für Studenten zu bestimmen.

**Methoden: **1427 Mini-CEX-Bewertungsformulare wurden von Medizinstudenten aus deren letztem Studienjahr gesammelt. Das schriftliche Feedback, das sich auf Stärken und Schwächen der Studierenden bezieht, wurde kategorisiert und mit den Variablen der Komplexität der klinischen Fälle, der klinischen Position der Gutachter und der klinischen Leistungsbewertung der Studierenden korreliert.

**Ergebnisse: **Die Anzahl der allgemeinen Feedback-Kommentare bezüglich der Stärken und der Weiterentwicklung der Studenten betrug 953 (65,3%) bzw. 604 (38,64%). Weniger als 30% der Rückmeldungen zu jeder Domäne wurde als spezifisches Feedback kategorisiert. Signifikante Zusammenhänge wurden zwischen dem Feedback zu den Stärken eines Studenten und der Komplexität der klinischen Fälle (Χ^2^=17,48, *p*<0,01) sowie der klinischen Position der Gutachter (Χ^2^=37,10, *p*<.01) gefunden. Ebenfalls bestand ein Zusammenhang zwischen dem Feedback zur Weiterentwicklung der Studierenden und der klinischen Position der Gutachter (Χ^2^=27,22, *p*<0,01).

**Schlussfolgerung: **Basierend auf den Mini-CEX-Formularen der Studentengruppe, die in dieser Studie untersucht wurde, kann man schließen, dass das in den Mini-CEX Bewertungsformularen abgegebene schriftliche Feedback allgemeiner Natur und wenig spezifisch war. Dieser Befund zeigt auf, dass klinische Lehrer besser für die Bereitstellung von Feedback ausgebildet werden müssen.

## 1. Introduction

The Mini Clinical Evaluation Exercise (Mini-CEX) [1] was originally developed for postgraduate medical training to allow an evaluation of a trainees’ ability to perform in real-time clinical practice. It was developed to replace traditional clinical evaluation exercises, such as long cases, which had limitations. These limitations included the number and variety of cases, length of time required for full assessments, complexity of the clinical setting and the difficulty of finding assessors willing and able to spend the time required [1], [2]. Despite the growing use of Mini-CEX’s in the undergraduate medical education setting [3], [4], [5], there has been little research examining their utility in this setting particularly in regard to the provision of feedback.

According to the social sciences and medical education literature, feedback is best described as “specific information about the comparison between one’s performance and a standard in order to improve the learners’ performance” [[6], p. 189]. In an extensive review of research evidence on the effectiveness of feedback, Hattie and Timperley concluded that effective feedback will help learners in answering three questions: where one is going, how one is going, and where to next [7]. Therefore, feedback should go beyond the outcome of a particular learning process. 

Embedded in the Mini-CEX format is the direct observation of students performing particular skills and the immediate and specific feedback provided following the observation, according to a predetermined assessment form. All of these features of the Mini-CEX are aligned to the characteristics of effective feedback [8], [9], which includes the timely provision of feedback to a recipient who is expecting it. Feedback should also be specific, derived from the observation and focus on remediable actions. In addition, students need to have the opportunity to clarify the feedback. This is in line with the practice of Mini-CEX where students have the opportunity to read the feedback and ask for questions or clarifications regarding the feedback. 

Apart from one study by Norcini, et al. [2] that demonstrated significant improvement of the scores in the Mini-CEXs over several encounters, there has been little research on the quality of feedback given to undergraduate students. There are also various opinions regarding the perceptions of students of the Mini-CEX, some of whom value it [4], [10], [11], and some who still consider it as a routine procedure with little effect on learning [12], [13].

Holmboe, Yepes, Williams, & Huot categorized feedback into four hierarchical groups (from the least to the most effective): giving recommendations, enabling learner reaction, asking for self-assessment and agreeing on an action plan [14]. They found in their study that most feedback provided during Mini-CEX encounters fell into the least effective (giving recommendations) category [[14]. Lack of experience and training, discomfort of the assessors, and inadequate time are some factors proposed to be the causes of the lack of these types of feedback [3], [14]. This situation may significantly diminish the usefulness of the Mini-CEX as a learning tool. 

Through the implementation of Mini-CEX, students are exposed to a variety of cases in various clinical settings and most importantly to opportunities to receive feedback over time. However, the small effect of the Mini-CEX on learning may plausibly stem from the poor quality of the feedback provided within the Mini-CEX [3], [12], [13], [14]. Therefore, in order to advance our understanding of the quality of written feedback provided in the Mini-CEX encounters, we analysed assessment forms from a cohort of final year medical students at a large Australian medical school. 

## 2. Method

**Figure 1:**
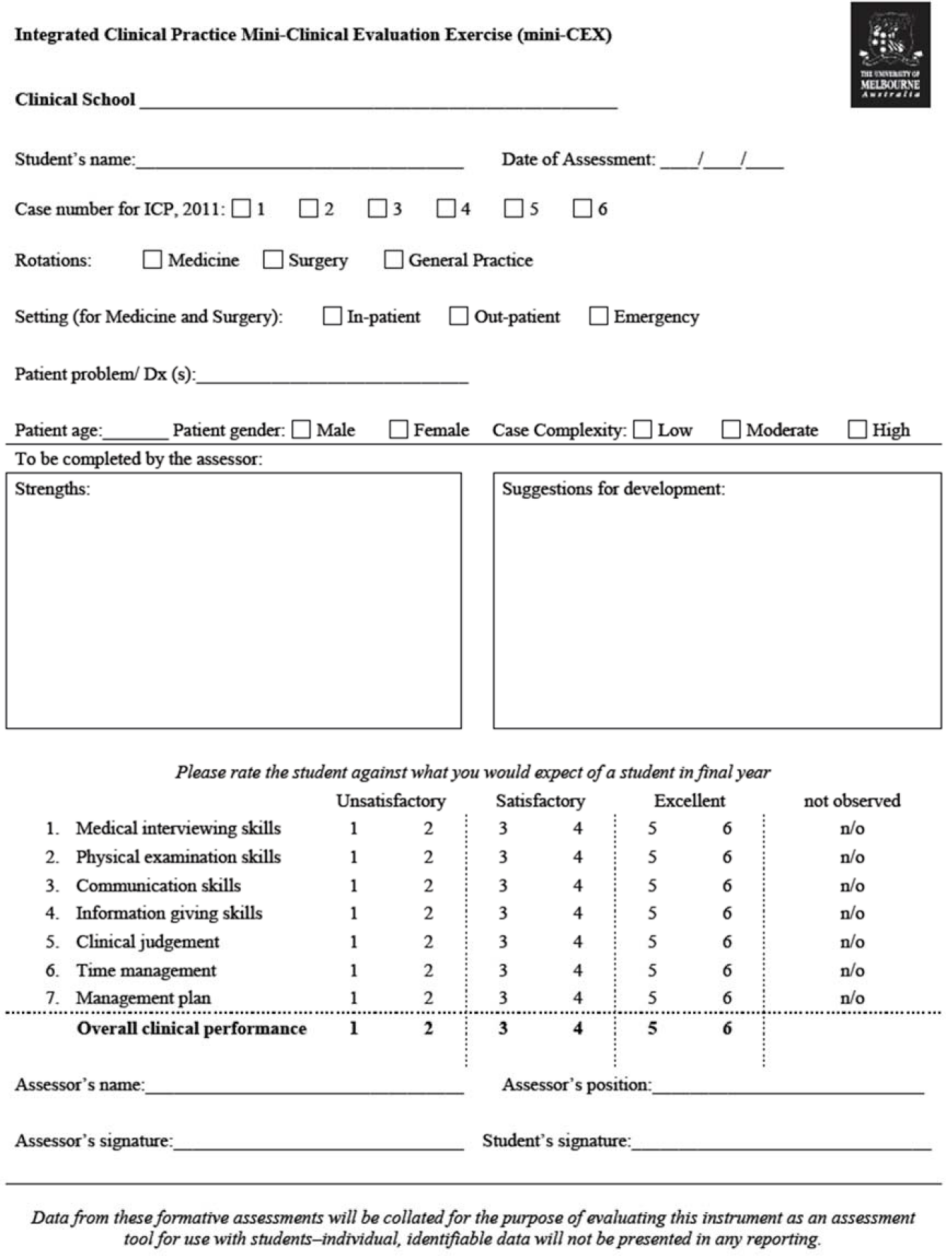
Mini-CEX assessment form used in the MBBS course

At the end of the final semester of a large Australian medical school MBBS (Bachelor of Medicine, Bachelor of Surgery) course, all Mini-CEX assessment forms at three of the five metropolitan clinical schools were collected (the form used in the course is provided as Figure 1 (Fig. 1)). The Mini-CEX is a hurdle requirement for passing the subject and each student was required to submit at least six completed Mini-CEX assessment forms (two forms for each of the three clinical terms; medicine, surgery, and general practice). The study was approved by the University Human Research Ethics Committee. 

Quantitative data were analysed using IBM SPSS version 19 (IBM Corp.). The assessor clinical positions were classified as consultant, registrar/fellow and doctor in training (resident/intern), according to the position hierarchy of hospital staff in the Australian medical context, and also general practitioner. Training sessions on Mini-CEX for assessors was provided at each clinical school but there is likely to have been some variation in the approach to these sessions taken by individual leader. Instructions on how to use the Mini-CEX assessment form, including the level of student performance, were provided for the assessors.

The coding manual for the written feedback categorization was developed based on Holmboe et al’s categories of feedback (giving recommendations, enabling learner reaction, asking for self-assessment and agreeing on action plan) [14] using a subset of Mini-CEX assessment forms. After analysis of a sub-group of responses, categories were collapsed to a dichotomous variable of general or specific feedback. 

Each entry of specific feedback was further categorized into the corresponding clinical performance domain. The suggestion for development was categorized into three main categories: general, specific, and feedback that provides more than just suggestions. Similar to feedback for students’ strengths, a specific suggestion was also classified into a suitable clinical performance domain. We conducted a content analysis [15] to determine the appropriate category for each feedback entry on the assessment form. 

Following this process, an independent rater was asked to categorize the feedback from 10% (n=140) randomly selected Mini-CEX assessment forms, to examine the inter-rater agreement using the two-way random model of intra-class correlation (ICC) analysis [16]. Only the main categories of feedback (no feedback, general and specific feedback) were included in the ICC analysis since the focus was on whether the feedback was general or specific, rather than on the clinical performance domain. When there was more than one category of feedback within a comment, only the highest category was included in the analysis. For example if a comment consisted of both general and specific feedback, we took the higher category of feedback (specific feedback) to represent the comment. The ICC coefficients for the feedback on students’ strength and development categories were .793 (p<.001, 95% CI=.721-.847) and .946 (p<.001, 95% CI=.961-.980) respectively. Both ICC coefficients were considered as good [17], which indicated a relatively high and substantial agreement between the raters. 

## 3. Results

One thousand four hundred and twenty-seven Mini-CEX assessment forms of final year medical students were collected. Table 1 (Tab. 1) provides a complete description on the background data of the Mini-CEX assessment forms and also the clinical cases used in those assessments. 

### 3.1. Feedback data on clinical performance

Each Mini-CEX assessment form had more than one type of feedback about the students’ strengths; therefore the total number of comments exceeded the number of forms. The application of the coding manual revealed that 65.3% feedback on students’ strengths was general feedback (as shown in Table 2 (Tab. 2)). In addition to that, 16.6% assessors left the feedback column blank. 

Six hundred and four (38.64%) written comments for students’ development were in the form of general suggestions (as shown in Table 3 (Tab. 3)). There was only one feedback comment which fitted into the category of feedback that provides more than suggestions. 

There were 211 (14.8%) Mini-CEX assessment forms that had neither feedback on students’ strengths nor suggestions for development. Thirty-one forms only contained some suggestions for students’ development. Within the 239 forms that consisted only of feedback on students’ strengths, more than 50% of those comments were categorized as general feedback. 

A contingency table of the relationship between clinical case complexity and feedback on students’ strength (no feedback, general and specific feedback categories) was developed. There was a significant association between feedback and clinical case complexity (Χ^2^=17.48, *p*<.01). The examination of Adjusted Standardized Residual (ASR) values, to identify the sub-categories contributing to the significant associations, revealed a significant association between high complexity case and no feedback (ASR=1.96). It can be interpreted that when the complexity of the clinical case used for the Mini-CEX was high, then significantly fewer assessors than expected did not give feedback. 

A recoding was done to incorporate “feedback that was more than suggestions” group into the category of “specific suggestions”, because there was only one comment of feedback that was more than suggestions, therefore the chi-square and Fisher’s exact test was not useful [16]. The chi-square test for the clinical case complexity and feedback for development (blank column, no suggestion, general and specific suggestion categories) contingency table demonstrated a non-significant association (Χ^2^=10.43, *p*>.05). 

For the purpose of a chi-square analysis, the categories for assessors’ clinical position were reduced (consultant, GP and registrar or fellow). There was a significant association between feedback on students’ strength and assessor clinical position (Χ^2^=37.10, *p*<.01). The examination of ASR values revealed a significant association between consultant and specific feedback and also between GP and specific feedback (ASR=1.96). These findings indicated that when the assessor was a consultant, significantly fewer assessors than expected provided feedback on students’ strength. On the other hand, when the assessor was a GP, significantly more assessors than expected delivered specific feedback.

The assessor clinical position was found to have a significant association with the feedback for students’ development (Χ^2^=27.22, *p*<.01). The examination of ASR values revealed a significant association between consultant and specific suggestion, GP and no suggestion, and registrar/fellow and no suggestion (ASR=1.96). If the assessors were consultants, then significantly more assessors than expected provided students with specific suggestions for development. When the Mini-CEX assessor was a registrar or fellow, significantly fewer assessors than expected provided no suggestions. This was in contrast to the findings related to GP in which significantly more GP than expected did not deliver feedback for students’ development. 

Further analysis using Spearman correlation test on the relationship between the ratings of students’ clinical performance and the feedback indicated that there were no significant relationships between feedback on students’ strengths and each domain of clinical performance. For every domain of clinical performance listed in the Mini-CEX form there was a negative weak significant relationship with the feedback provided for students’ development (r value ranging from -.22 to -.28, p<.01). The higher the rating for a particular domain of clinical performance, the less likely it was for the feedback to be specific. However, these significant relationships need to be treated with caution due to the large sample. 

#### 3.2. Ratings of students’ clinical performance

The results demonstrated that of the 1427 assessment forms analyzed, there were no assessors who gave a student a rating of one for overall clinical performance. The minimum rating obtained in almost all domains was three (satisfactory), with the mean score of each domain ranged from 4.53 to 4.77 (SD ranged from .814 to .728). Only within the time management domain was there a rating of two, although the mean score was still 4.56 (*SD*=.779). 

Relationships among the domains of clinical performance revealed moderate to strong positive significant relationships (r value ranging from .59 to 79). This finding indicated a tendency to give similar rating for every domain being assessed for a particular student. 

## 4. Discussion

Results show that most feedback provided in the Mini-CEX assessment forms was either general feedback on students’ strength or general suggestions. Less than 30% of the feedback comments was directed to improve students’ performance in a specific way, and the number of specific feedback comments aimed to ensure good practice was even lower. When correlated with variables related to the provision of feedback such as assessors clinical position, clinical case complexity and ratings of students’ clinical performance, there were several significant associations found.

Most of the written feedback, either on students’ strength or for their development, was categorized as general feedback. If the feedback categories from Holmboe, et al. [14] were applied, then there was only one feedback comment that would be considered as category four (“agreeing on action plan”). The rest of the written feedback was categorized as category one (“giving recommendations”). Fernando, et al. [3] and Jackson and Wall [12] also found that there are still problems in providing feedback that is more than just suggestions. 

Feedback within the Mini-CEX needs to focus not only on the outcome of a task, but more importantly it needs to be aimed towards the learning process behind the task. Balzer, Doherty and O’Connor [18] discussed the differences between outcome feedback and cognitive feedback, in which the latter is preferable. In delivering feedback it is essential to make the learner the focus of attention, since it will likely increase feedback receptivity and usefulness [19], [20], [21], [22]. Therefore, it is important to acknowledge students’ needs and responses during feedback provision in the Mini-CEX and students need to be assisted in trying to identify their own deficiencies and strengths. 

There were some Mini-CEX forms that did not contain any feedback or contained either only general feedback on students’ strength or suggestions for development, although the number of these was low. This warrants further attention since one of the important aims of Mini-CEX is to provide immediate feedback to the students. Assessors also need to understand that both positive and negative feedback is very important. Balance between feedback on students’ strengths and feedback for students’ development is essential to increase the usefulness of feedback and students’ receptivity upon it [23]. 

The present study found that the higher the rating of students’ clinical performance, the less likely for the feedback for students’ development to be specific. The assessors might consider that students with high scores in their Mini-CEX do not need any more feedback for their own continuous development. This is a perspective that needs to be modified, since every performance of a student requires feedback, whether to correct mistakes, maintain positive aspects or even further develop them. Providing feedback for each student performance will support the function of Mini-CEX as a learning tool. The analysis also demonstrated that when the clinical case was highly complex, it was more likely for the assessors to provide feedback on students’ strengths. However, there was no significant association between the case complexity and the feedback on students’ development.

More varied results were found in relation to association of the assessors’ clinical position with the feedback provided. Consultants were more likely to give specific feedback for development, but they tended to provide less specific feedback on students’ strengths. General practitioners are more likely to deliver a higher amount of specific feedback on students’ strengths. On the other hand, they are less likely to provide feedback for students’ development. Interestingly, registrars or fellows, who are not expected to be Mini-CEX assessors, are more likely to give feedback for students’ development. Proper training for registrars or fellows in the area of medical teaching and assessment might be useful to improve their ability to evaluate students and provide adequate feedback. 

Fernando, et al. [3] used different category of assessors when analysing the association between feedback and type of assessors based on 396 Mini-CEX sessions. Despite the weak significance and limited generalizability, the possibility for academic trainees (teaching and research fellow) to give suggestions for development and action plans was higher compared to consultants and clinical trainees. They argue that familiarity with teaching and assessment principles, including how to give feedback, is the possible cause. These findings further corroborate the need for a structured assessors training to standardize their ability in delivering feedback.

The distribution of Mini-CEX assessments in the present study still indicated an imbalance, for example there were only about 3% of Mini-CEX assessments in the emergency setting in either medicine or surgery rotation. Undergraduate medical students have a shorter rotating period in each clinical attachment and limited exposure to clinical cases, compared to the postgraduate trainees for whom the Mini-CEX was first designed. Therefore, it might be beneficial if a more structured Mini-CEX is provided to students, including the selection of clinical cases and settings by teachers. 

The medical school expects the assessors to be consultants or GPs, however there are still doctors in training who serve as the Mini-CEX assessors. A similar situation also occurred in the study by Fernando, et al. [3]. Variability of assessors is in one respect an important aspect of Mini-CEX in order to obtain diverse perspectives regarding a student performance, but on the other hand, there needs to be a system to ensure that every assessor has the knowledge about the level of performance expected from the students and the ability to produce a valid and reliable score. 

There are a number of possible explanations for the high ratings obtained by students. The Mini-CEX at this particular university is used as a hurdle assessment. Each student needs to submit six Mini-CEX assessment forms in a semester and can select the best six of several Mini-CEX the student has experienced. The high ratings can also be caused by a tendency of the assessors to avoid giving low scores for students. In the study investigating Mini-CEX in an undergraduate setting, Hill and Kendall found that assessors were quite hesitant to give low ratings if they were on their own, specifically because they could not get a confirmation from another assessor about the accuracy of their rating and also because they were required to deliver feedback immediately afterwards [4]. Based on a review of the research on Mini-CEX from 1995-2009, Hawkins, Margolis, Durning and Norcini concluded that high ratings in Mini-CEX were common and one of the plausible causes was the assessors’ concern on the impact of their ratings on students [24]]. 

The complexity of the clinical case was not related to the rating of students’ clinical performance. However, the seven domains of clinical performance were correlated. This finding indicates that to some extent the rating may not be an independent measure of each domain of clinical performance. Hill, Kendall, Galbraith and Crossley [25] and Margolis, et al. [26] identified very strong positive relationships between domain scores and they might hinder the ability of Mini-CEX to assess an individual domain of competency. Hawkins, et al. argued that it might be difficult for the assessors to break down students’ performance into several domains or the domains themselves might be correlated [24]. 

The moderate to strong positive relationships obtained in the present study suggests that to some extent the scoring system may also be useful to discriminate students’ performance among several components, or in this particular batch of students, there were quite a high number of students demonstrating significantly good or poor performance in a particular domain. This situation may lead to a positive relationship that is not as strong as the one identified in other studies. 

## 5. Conclusions

The function of Mini-CEX as a learning tool lies in its ability to provide an opportunity for direct observation and useful feedback to medical students. However, the present study reveals that the function of Mini-CEX to provide specific written feedback is yet to be achieved. Our findings suggest that the concept of student-centred feedback needs to be emphasized throughout the feedback provision process, without ignoring the fact that clinical case complexity and the assessors’ clinical position are likely to influence the type of feedback provided. Structured training for clinical teachers and Mini-CEX assessors to improve the ability to provide specific and student-centred feedback is then important to optimize the learning tool of Mini-CEX. 

## Competing interests

The authors declare that they have no competing interests. 

## Figures and Tables

**Table 1:**
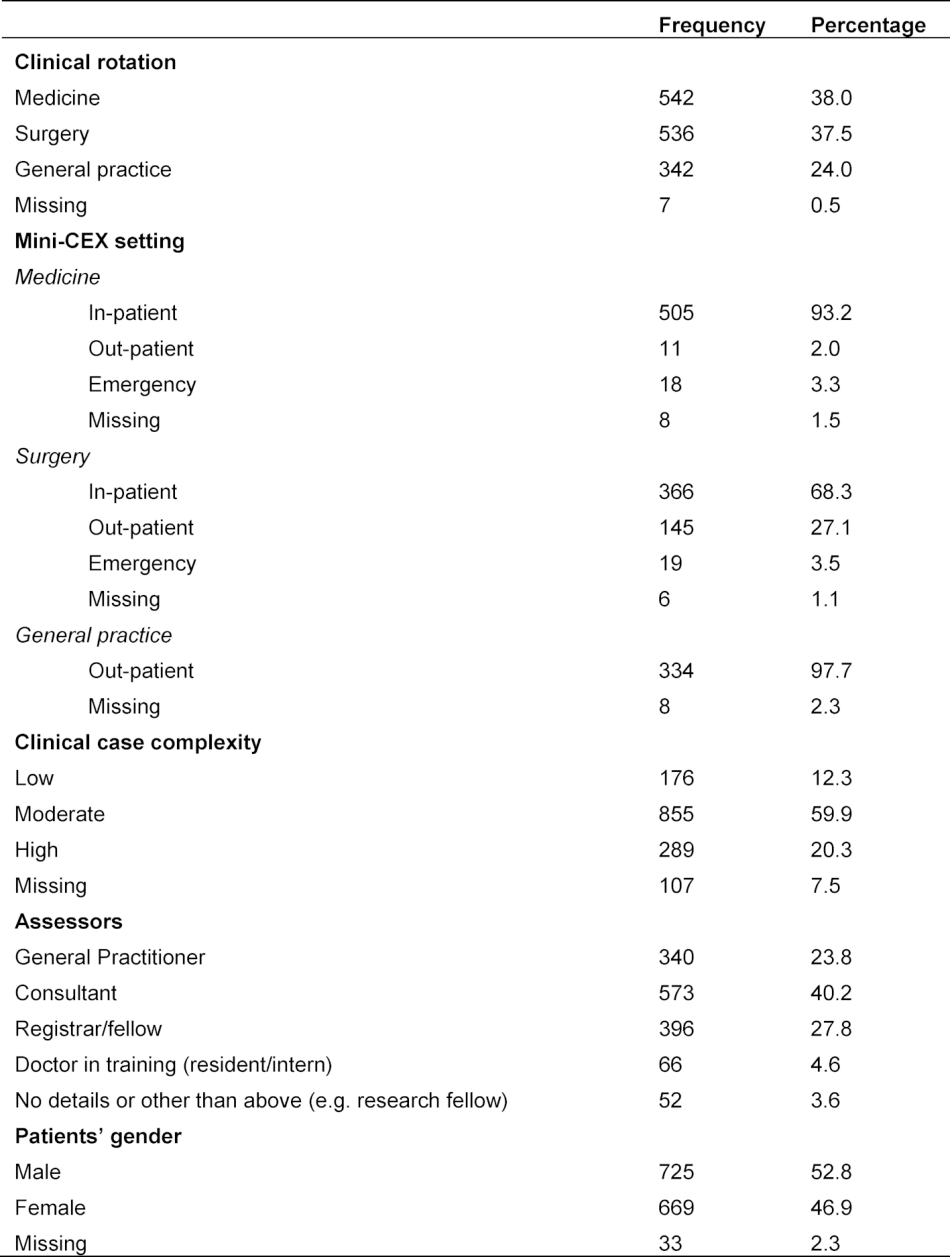
Background data of the Mini-CEX assessment forms

**Table 2:**
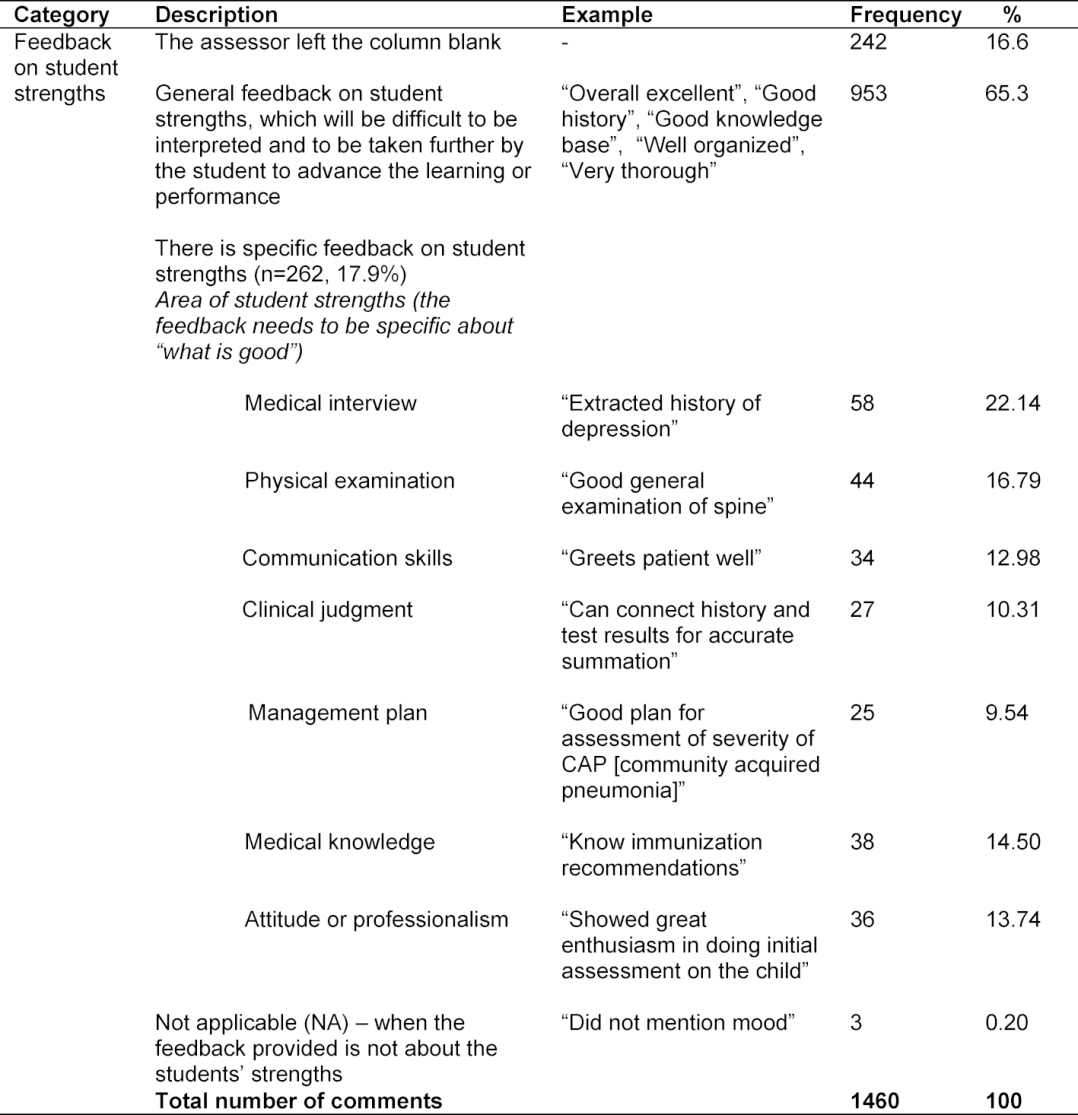
Categorization of feedback on students’ strengths

**Table 3:**
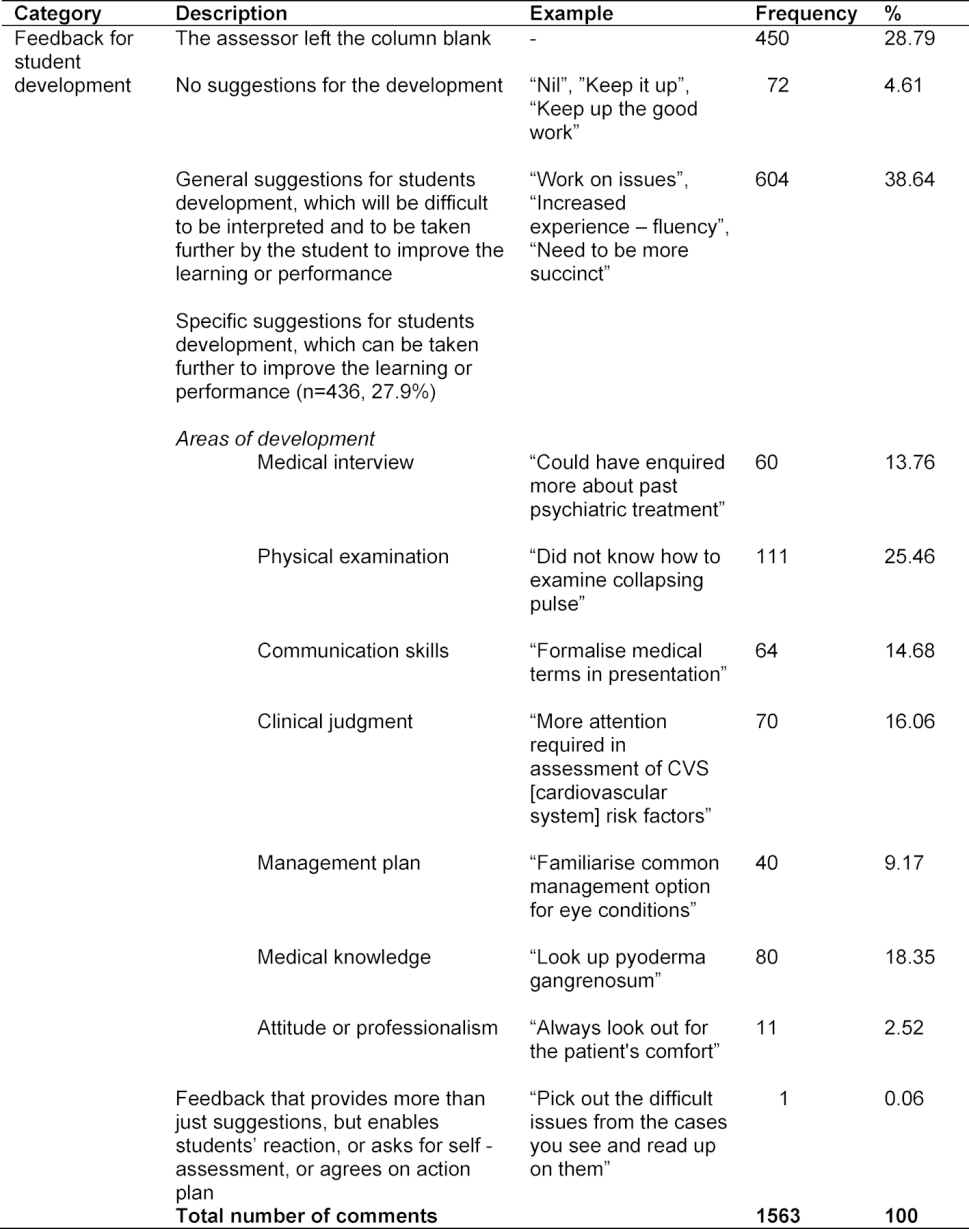
Categorization of feedback for students’ development
